# Integrated physiological, multi-omics analyses reveal key factors underlying seed abortion in *Dimocarpus longan*

**DOI:** 10.3389/fpls.2026.1778131

**Published:** 2026-03-02

**Authors:** Hong-ye Qiu, Xian-quan Qin, Chen Fang, Yan-jie Hou, Dong-bo Li, Jing-yi You, Ning Xu, Xiaolin Cai, Hongli Li

**Affiliations:** Horticulture Research Institute, Guangxi Academy of Agriculture Sciences, Nanning, China

**Keywords:** boron deficiency, embryo abortion, longan (*Dimocarpus longan*), nutrient imbalance, proteomics, transcriptomics

## Abstract

Embryo abortion severely limits fruit set and yield stability in longan (*Dimocarpus longan*), yet the upstream physiological triggers and coordinated molecular program remain incompletely defined. Here, we characterized normal seed-forming (NF) and aborted seed-forming (AF) fruits at the critical abortion window by integrating phenotyping, mineral nutrient profiling, embryo-targeted RNA sequencing, and quantitative proteomics, followed by cross-omics association analyses. Orchards with high abortion incidence exhibited markedly low available boron, and aborted embryos displayed a distinctive nutrient-partitioning pattern characterized by severe embryonic boron depletion despite broad changes in other elements. Transcriptome analysis identified 3,865 differentially expressed genes (1,993 upregulated and 1,872 downregulated in AF), with enrichment in pathways related to phenylpropanoid biosynthesis, starch and sucrose metabolism, amino sugar and nucleotide sugar metabolism, plant hormone signal transduction, and MAPK signaling. Quantitative proteomics revealed 1,518 differentially accumulated proteins (342 increased and 1,176 decreased in AF), highlighting a global trend toward reduced protein abundance in aborted embryos. Integrated transcriptome–proteome analysis detected 374 shared features with strong concordance between mRNA and protein fold changes (93.6% concordant; r = 0.82), reinforcing a coordinated regulatory program at the abortion stage. Across datasets, embryo abortion was associated with disrupted boron-related cell wall processes, altered carbohydrate transport and mobilization, extensive hormone/MAPK rewiring, and pronounced repression of chloroplast-associated programs including photosynthetic light reactions and pigment/tetrapyrrole metabolism, coupled with redox and energy imbalance. qRT-PCR of eight mechanism-anchored candidates supported RNA-seq trends. Together, these results support a model in which embryonic boron depletion and impaired cell wall integrity are associated with, and may contribute to, a cascade of metabolic and signaling reprogramming that culminates in embryo growth arrest and degeneration, providing actionable markers and targets to improve seed development and fruit set in longan.

## Introduction

*Dimocarpus longan* Lour. (longan) is a high-value subtropical fruit tree widely cultivated in southern China and Southeast Asia. Fruit set and commercial yield depend on successful fertilization and subsequent seed/embryo development that supports pericarp growth and fruit retention. However, many production orchards experience substantial losses due to a recurrent disorder characterized by early embryo degeneration, aborted seeds, and small, low-quality fruits. Such reproductive failure directly reduces yield stability and increases production risk.

Embryo abortion is increasingly recognized as a regulated developmental outcome rather than a purely stochastic failure. Across crops and perennial fruit trees, reproductive tissues can abort at multiple stages in response to internal resource constraints, hormonal imbalances, and environmental stress, representing a developmental decision that reallocates resources to surviving sinks (Saini et al., 2025). In fleshy fruit, embryo viability is particularly sensitive to maternal–filial nutrient allocation across seed interfaces, carbohydrate delivery and sink establishment, and stress signaling networks that integrate reactive oxygen species (ROS) with hormone pathways (Du et al., 2023; Xie et al., 2024). Nevertheless, the dominant triggers and core molecular programs underlying longan embryo abortion remain poorly resolved.

Mineral nutrition is central to early embryogenesis. Among micronutrients, boron (B) has an exceptional role in reproductive development because its best-established biochemical function is the formation of borate diester cross-links between rhamnogalacturonan-II (RG-II) domains of pectin, thereby stabilizing primary cell wall architecture and wall–membrane attachment (Camacho-Cristóbal et al., 2008; Begum and Fry, 2023; Hays et al., 2025). B deficiency can therefore compromise cell division, tissue cohesion, and developmental patterning during the rapid growth phase of young embryos. B homeostasis is mainly controlled by BOR exporters and NIP aquaporin-like channels, and cultivar-specific expression of these transporters has been linked to B utilization and reproductive performance (Wang et al., 2024). In fruit trees, B supply and B-mediated metabolic reprogramming can markedly affect fruit set and early development, supporting the notion that B status is a plausible upstream determinant of embryo viability (Michailidis et al., 2023; Vera-Maldonado et al., 2024).

Carbohydrate supply constitutes another major bottleneck for embryo survival. Developing embryos depend on maternal sucrose delivered through the seed coat and endosperm, which requires coordinated activity of membrane transporters and apoplastic/ symplastic conversion steps (Chen et al., 2015; Keller and Neuhaus, 2025). SWEET-family sugar transporters have emerged as critical determinants of source–sink allocation and seed filling, and their disruption can lead to reduced embryo growth and seed abortion in multiple systems (Breia et al., 2021). Consistent with this, constrained assimilate availability is sufficient to trigger kernel abortion in cereals and is generally associated with shifts in sugar metabolism and sink strength (Shen et al., 2018). In addition, growth–defense trade-offs frequently redirect carbon from biomass formation toward secondary metabolism such as phenylpropanoid pathways, which can further limit embryonic growth under stress (Yang et al., 2025).

Hormone signaling and stress-response networks provide the regulatory layer that integrates nutrient and carbon status into developmental outcomes. Auxin gradients and polar auxin transport are essential for embryo patterning and organogenesis, and disruption of auxin biosynthesis/transport/response typically produces severe embryogenesis defects (Verma et al., 2021). Abscisic acid (ABA), gibberellins (GA), ethylene-related regulators (AP2/ERF), and MAPK signaling pathways mediate stress adaptation and growth restraint, often acting together with ROS signaling modules. A growing body of evidence indicates that ROS overproduction and oxidative signaling can be causal in abortion phenotypes, including recent work demonstrating DNA methylation–mediated ROS elevation as a contributor to seed abortion in litchi (Xie et al., 2024). These observations suggest that embryo abortion in longan may reflect a coordinated cascade in which nutrient misallocation and carbon limitation activate hormone/MAPK/ROS circuits and reprogram metabolism.

Mechanistic resolution of such complex traits benefits from synchronized multi-omics. Transcriptomics can identify upstream regulatory shifts, whereas proteomics captures post-transcriptional buffering and the functional proteome closer to phenotype. Multi-omics studies in fruit trees and other species have highlighted embryo-abortion–associated signatures involving carbohydrate metabolism, hormone signaling, stress responses, and energy pathways, but the degree to which mRNA changes translate into protein-level changes varies by system and developmental stage (Qiu et al., 2020; Shao et al., 2022; Du et al., 2023). For longan, a systems-level analysis that integrates field-relevant physiology, tissue mineral profiles, embryo-targeted RNA-seq, and quantitative proteomics at the critical abortion window has remained limited.

Here, we address this gap by integrating phenotypic characterization to define the critical abortion stage, mineral nutrient profiling across maternal tissues (leaf and pericarp) and embryos, embryo-focused transcriptomics (RNA-seq) and quantitative proteomics (TMT-based LC–MS/MS), and cross-omics association analyses to identify conserved, concordantly regulated pathways. This study aims to delineate the physiological basis and core molecular network of longan embryo abortion and to prioritize candidate targets for diagnosis and improvement of fruit set.

## 2 Materials and methods

### 2.1 Plant materials and sampling

**Figure 1:**
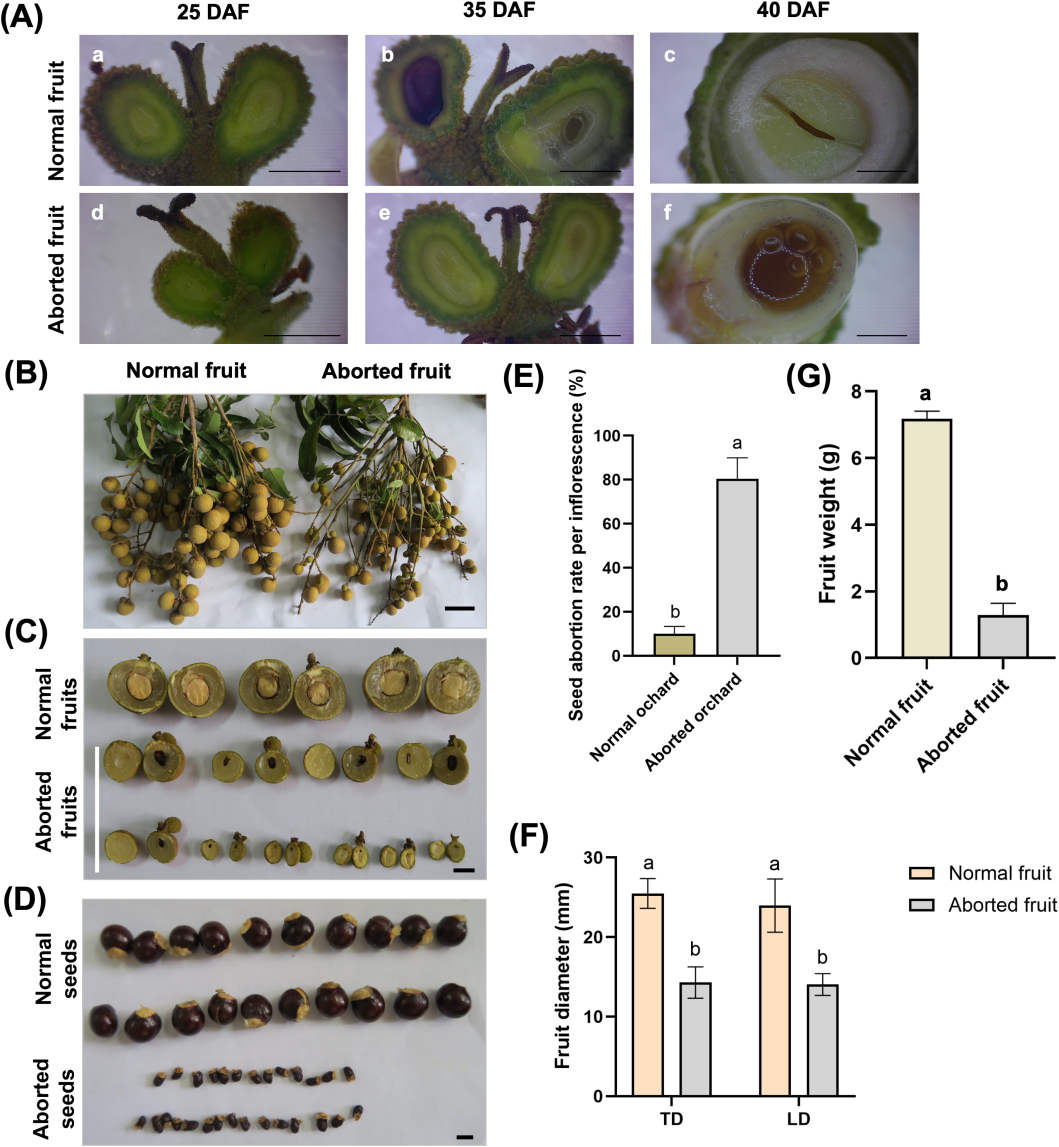
Phenotypic comparison between normal and aborted fruits and associated differences in fruit traits. **(A)** Representative stereomicroscope images of normal seed-forming fruits (NF; (a–c), upper row) and aborted seed-forming fruits (AF; d–f, lower row) at different days after flowering (DAF). At 25 DAF (a, d), NF fruits show uniform ovary expansion and green pericarp with normally developing ovules, whereas AF fruits display reduced enlargement and early developmental arrest. At 35 DAF (b, e), NF fruits maintain normal seed development, while AF fruits exhibit visible ovule discoloration and degeneration, with browning/purpling indicative of abortion progression. At 40 DAF (c, f); transverse sections), NF seeds contain a compact, pale-yellow embryo with differentiated endosperm and an intact, translucent seed coat, whereas AF seeds show severe collapse of internal tissues with liquefaction/necrosis and loss of normal embryo structure. Panels (a–f) correspond to the labeled subpanels in the figure; images are representative of observations across multiple fruits. Panels b and e correspond to 35 DAF and were captured using the same imaging settings as the other timepoints; **(B)** Representative fruit bunches showing normal fruits versus aborted fruits; **(C)** transverse sections of normal and aborted fruits; **(D)** morphology of normal and aborted seeds; **(E)** seed abortion rate per inflorescence (%) in normal versus aborted orchards; **(F)** transverse diameter (TD) and longitudinal diameter (LD) of normal and aborted fruits; **(G)** fruit weight (g) of normal and aborted fruits. Bars represent mean ± SE, different letters indicate significant differences (P < 0.05). Scale bars: 1 cm in panels **(A)** (a, b, d, e) and panel **(B–D)**; 50 mm in panels **(A)** (c, f).

Normal (NF) and aborted (AF) seed-forming fruits of Dimocarpus longan cv. Shixia were collected from a commercial orchard in Baise, Guangxi, China (same orchard block) on the same sampling date. The critical abortion window was defined by stage-resolved morphological tracking across 25, 35, and 40 days after flowering (DAF) (**Figure 1A**) and confirmed by internal dissection of seeds/embryos. Three independent biological replicates were collected for each group; each replicate corresponded to one independently sampled tree (n = 3 trees per group). For each replicate, ten fruits were harvested from the same tree, and embryos were dissected under a stereomicroscope; embryos from the same replicate were pooled to form one composite sample for downstream mineral analysis, RNA-seq, and proteomics. Fruits were classified as NF or AF based on external phenotype criteria, and abortion status was confirmed by internal dissection (embryo collapse/browning and loss of normal endosperm/seed structure). All tissues (leaf, pericarp, embryo) were immediately frozen in liquid nitrogen and stored at −80 °C until analysis.

### 2.2 Mineral element analysis

For mineral profiling, embryo tissues were dissected under a stereomicroscope as defined in Methods 2.1 (embryo-containing seed fraction), oven-dried at 65 °C to constant weight, and ground to <0.5 mm. For each sample, 0.200 g of dry powder was digested with trace-metal grade HNO_3_ and H_2_O_2_ using a closed-vessel microwave digestion system (CEM MARS 6, USA) following the manufacturer-recommended plant tissue digestion program (target temperature 180 °C, hold time 20 min). Digests were brought to a final volume of 25 mL with ultrapure water and analyzed by ICP-OES (Agilent 5110, Agilent Technologies) to quantify Ca, Mg, K, Si, Fe, Mn, Zn, Cu, and other elements (Hansen et al., 2009). Total phosphorus was measured using the molybdate–ascorbic acid method after wet-acid digestion (Murphy and Riley, 1962). Total boron was determined by azomethine-H colorimetry (John et al., 1975). Total nitrogen was measured using the Kjeldahl digestion–distillation method (Kjeltec 8400, FOSS) (Bremner, 1960). Element concentrations are reported on a dry weight (DW) basis. Quality control included procedural blanks and a plant-matrix certified reference material (CRM; GBW(E)100348), with recoveries for measured elements within 90–110%.

### 2.3 Determination of soluble sugars and vitamin C

Soluble sugars were quantified by the anthrone–sulfuric acid method using glucose as the calibration standard (0–100 μg mL^-^¹) and absorbance at 620 nm. Ascorbic acid was extracted with 2% oxalic acid and titrated with 2,6-dichlorophenolindophenol (DCPIP) to a persistent light-pink endpoint. Results are reported as mg g^-^¹ FW for soluble sugars and μg g^-^¹ FW for vitamin C.

### 2.4 RNA extraction and sequencing

Total RNA was extracted from embryo tissues using a TRIzol-based protocol (Invitrogen) followed by DNase I treatment (Takara). RNA quality was assessed using an Agilent 2100 Bioanalyzer and NanoDrop ND-2000; samples with RIN ≥ 8.0 were used for library preparation. Poly(A)+ mRNA was enriched and libraries were constructed using the Illumina TruSeq RNA Sample Prep Kit. Libraries were sequenced on an Illumina NovaSeq 6000 platform to generate 150 bp paired-end reads.

### 2.5 Transcriptome data processing and differential expression analysis

Clean reads from each sample were aligned to the Dimocarpus longan reference genome (GigaDB dataset 100276; annotation set: longan.iprscan) using HISAT2 v2.1.0, and the mapping rate ranged from 85.48% to 90.98% across samples. Reference-guided transcript assembly was performed using StringTie (v2.2.1), and gene expression levels were quantified using RSEM (v1.3.3) and normalized as TPM. Differentially expressed genes (DEGs) was performed in R (v4.2.2) using DESeq2 (v1.38.3) with thresholds of |log_2_FC| ≥ 1 and adjusted P ≤ 0.05. GO enrichment and KEGG pathway enrichment were performed with adjusted P ≤ 0.05 as the significance threshold.

### 2.6 Protein extraction, TMT labeling, and LC–MS/MS analysis

Total proteins were extracted from frozen embryo tissues using urea/SDS lysis buffer containing protease inhibitors. Protein concentration was determined by the BCA assay (Thermo). Proteins were reduced (TCEP) and alkylated (iodoacetamide), precipitated, resuspended in 100 mM TEAB, and digested with trypsin (1:50, w/w) overnight at 37 °C. Peptides were desalted, quantified, labeled with TMT 10/11-plex reagents (Thermo Fisher), pooled, and vacuum-dried. Peptides were fractionated by high-pH reversed-phase chromatography (Vanquish Flex UPLC, Thermo) and analyzed by LC–MS/MS on an Evosep One system coupled to an Orbitrap Exploris 480 (Thermo) operating in data-dependent acquisition mode with TurboTMT.

### 2.7 Proteomic data processing and differential accumulation analysis

Raw MS data were processed using Proteome Discoverer (v2.4, Thermo Scientific) and searched against the *D. longan* protein database (GigaDB dataset 100276; annotation set “longan.iprscan”; protein accessions represented by *D. longan* gene model IDs such as “Dlo_xxxxx.x”) appended with a common contaminants database (cRAP). Reporter-ion intensities were quantified and normalized in Proteome Discoverer using median scaling (total peptide amount normalization), and protein abundance was reported as normalized abundance values generated during the Proteome Discoverer processing workflow. Differentially accumulated proteins (DAPs) were identified by comparing the two biological groups using three biological replicates per group (NF_1–NF_3 vs AF_1–AF_3). Statistical testing followed the Student’s t-test–based comparison used in the “Diff_student” output table, and P values were further adjusted for multiple testing using the Benjamini–Hochberg procedure. Proteins were considered differentially accumulated if they met FC(AF/NF) > 1.20 or < 0.83 together with q < 0.05. Functional annotation of identified proteins was performed using standard databases and versions employed in the analysis pipeline, including Pfam (v34.0), KEGG (v2021.09), eggNOG (v2020.06), Swiss-Prot (v2021.06), GO (v2021.0918), and NR (v2021.10). GO enrichment was conducted using goatools (v0.6.5), and KEGG enrichment was performed using Python-based scripts as implemented in the analysis workflow.

### 2.8 Integrated multi-omics analysis

Transcriptome–proteome integration was performed at the gene level using consistent *D. longan* gene identifiers. Protein accessions were mapped to gene IDs based on the same reference annotation used for RNA-seq. When multiple transcript isoforms corresponded to one gene, RNA-seq expression was summarized at the gene level as implemented in DESeq2. For proteomics, peptides mapping to protein groups were handled by protein-group inference in Proteome Discoverer with unique-peptide priority (i.e., protein groups supported by unique peptides were retained as the primary evidence). When multiple protein entries mapped to the same gene, a representative protein was selected using a hierarchical rule of (i) highest number of unique peptides, then (ii) highest sequence coverage, and if still tied (iii) highest summed (normalized) reporter-ion intensity; alternatively, when appropriate for downstream concordance plots, protein intensities were aggregated to the gene level by summing normalized intensities of proteins mapped to the same gene. “Shared” molecules were defined as genes detected in both datasets with quantifiable expression/intensity in ≥ 2 of 3 biological replicates per group; concordance analysis used log_2_ fold changes from DESeq2 (RNA) and log2 fold changes derived from normalized TMT reporter-ion intensities (protein). Significance thresholds followed the respective DEG/DAP criteria, i.e., DEGs defined by DESeq2 (FDR-adjusted P < 0.05) and DAPs defined by the proteomics differential analysis output (Student’s t-test P < 0.05 with fold-change threshold as applied in the DAP table).

### 2.9 qRT-PCR validation

Eight embryo-abortion–related candidate genes spanning five mechanistic axes were selected for qRT-PCR validation (**Supplementary Table S1**). Gene-specific primers were designed and qRT-PCR was performed using SYBR Green chemistry on a QuantStudio 5 Real-Time PCR System. Relative expression was calculated using the 2^−ΔΔCt^ method with ACTIN as the reference gene.

### 2.10 Statistical analysis

All physiological, mineral, transcriptomic, and proteomic analyses were performed using three independent biological replicates per group (n = 3), where each replicate represents an independent field unit as defined in Methods 2.1 (tree-level replicate). For comparisons between NF and AF, statistical significance was assessed using two-tailed Student’s *t*-test. Where multiple tissues or multiple traits were tested simultaneously, P-values were adjusted using Benjamini–Hochberg false discovery rate (FDR) or an appropriate multiple-testing correction, with P < 0.05 considered significant unless otherwise stated.

## 3 Results

### 3.1 Phenotypic characterization and critical stage of embryo abortion

Field observations and morphological analyses revealed pronounced differences in fruit development between normal seed-forming (NF) fruits and aborted seed-forming (AF) fruits. NF fruits developed uniformly (**Figures 1A, B**), with symmetrical ovary expansion and persistent green coloration of both pericarp and seed tissues during early development. Cross-sections showed well-developed embryos with compact yellowish kernels, differentiated endosperm, and an intact, translucent seed coat (**Figure 1C**, upper row; 40 DAF).

In contrast, AF fruits exhibited arrested development shortly after fertilization. Ovaries displayed reduced enlargement and early discoloration, accompanied by progressive browning or purpling of ovules (**Figure 1C**, lower row), indicative of degenerative processes. In severely affected fruits, the embryo sac collapsed, nucellar tissue darkened, and seed coat differentiation was absent. By later stages (**Figure 1D**), AF seeds showed pronounced shrinkage, tissue liquefaction, or complete necrosis, whereas NF seeds remained plump and structurally intact.

Quantitative traits further corroborated these morphological defects. AF fruits exhibited a significantly higher seed abortion rate compared with NF fruits (**Figure 1E**), confirming the consistency of the phenomenon across sampled individuals (n = 44). Fruit size in AF was markedly reduced, showing a compressed distribution far below those of NF (**Figure 1F**). Similarly, fruit weight in AF was only a fraction of that in normal fruits (**Figure 1G**), reflecting profound developmental arrest associated with embryo failure.

Integration of anatomical and quantitative evidence indicates that embryo abortion is initiated during the early seed-forming stage, when the fertilized ovule transitions into a functional developing seed. Disruption at this stage impairs embryo enlargement and endosperm formation, subsequently limiting pericarp expansion and leading to small, light, and structurally degraded fruits. These findings identify early embryogenesis as the critical developmental window determining seed set and fruit retention in longan.

### 3.2 Systemic nutrient redistribution and severe embryonic boron deficiency

**Figure 2:**
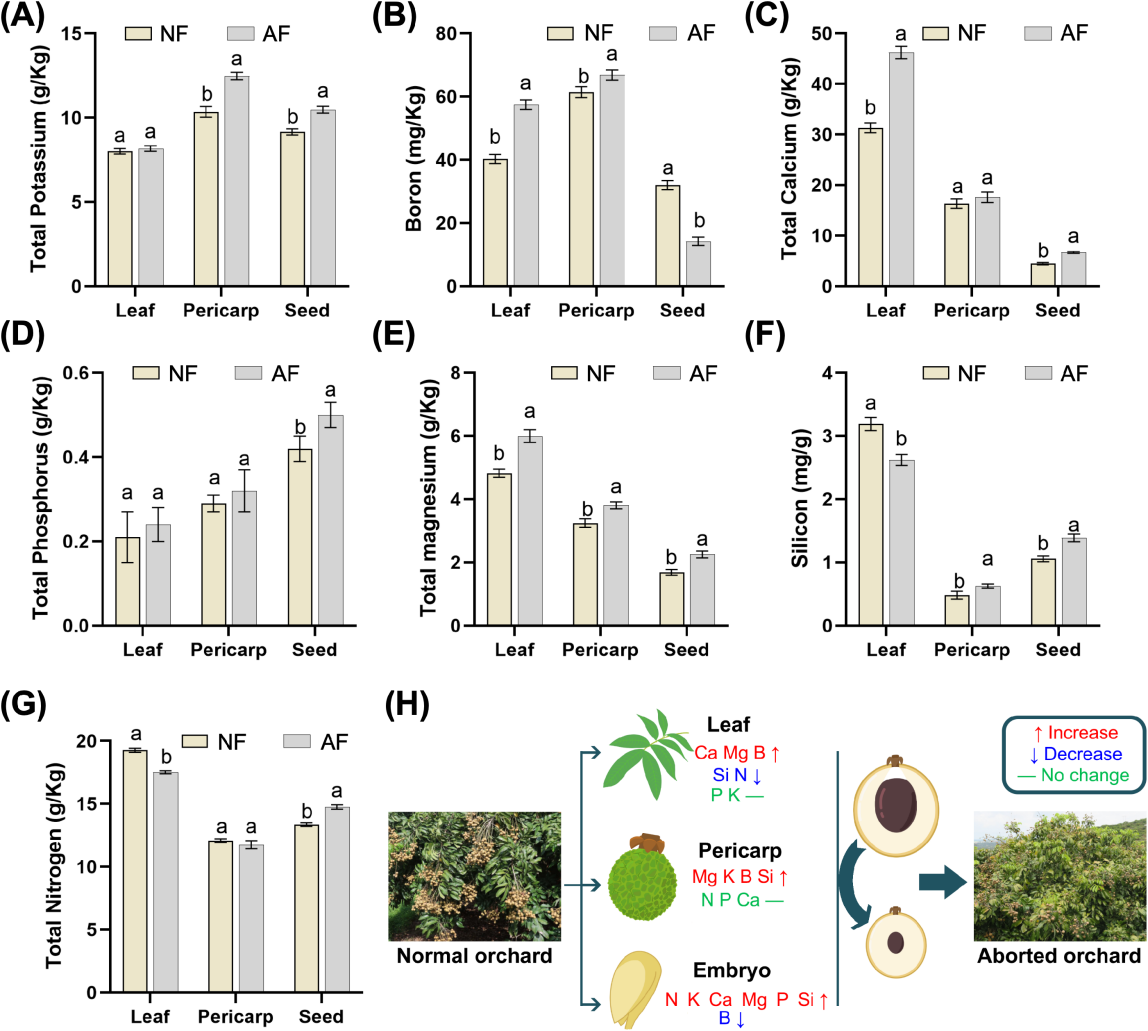
Comparative mineral nutrient profiles in leaf, pericarp and seed/embryo tissues between Normal seed fruit and Aborted seed fruit. **(A)** total potassium (g/kg); **(B)** boron (mg/kg); **(C)** total calcium (g/kg); **(D)** total phosphorus (g/kg); **(E)** total magnesium (g/kg); **(F)** silicon (mg/g); **(G)** total nitrogen (g/kg); **(H)** schematic summary illustrating tissue-specific nutrient changes from normal to aborted orchards/fruits, with arrows indicating increase, decrease or no change; bars represent mean ± SE; different letters denote significant differences (P < 0.05).

Mineral element profiling revealed pronounced tissue-specific nutrient redistribution between normal seed-forming (NF) and aborted seed-forming (AF) fruits at the critical abortion stage (**Figures 2A–G**). In leaves, trees producing AF fruits showed higher concentrations of boron (B), calcium (Ca), magnesium (Mg), potassium (K), and phosphorus (P) than the NF group, whereas silicon (Si) and nitrogen (N) were lower (**Figures 2B–G**). In the pericarp, AF fruits also showed higher K, B, Ca, Mg, P, and Si than NF fruits (**Figures 2A–F**). In the embryo (see Methods 2.1–2.2 for dissection definition), the nutrient pattern was distinct: AF embryos displayed higher K, Ca, Mg, P, Si, and N than NF embryos, while B was markedly reduced, supporting an embryo-associated B depletion phenotype linked to abortion rather than a uniform reduction across all elements. Consistent with these measurements, the schematic summary highlights a cross-tissue mismatch in boron distribution, with boron increased in maternal tissues but decreased in the embryo, suggesting impaired boron allocation to filial organs (**Figure 2H**).

Fruit quality traits also differed between groups (**Supplementary Figure S1**). Soluble sugar content was significantly higher in normal fruits (94.15 ± 25.23 mg g^-^¹ FW) than in aborted fruits (50.08 ± 1.30 mg g^-^¹ FW). Vitamin C content showed the same direction, with normal fruits (176.15 ± 36.21 μg g^-^¹ FW) exceeding aborted fruits (71.61 ± 16.14 μg g^-^¹ FW) (different letters indicate P < 0.05). Together, these results identify embryo-specific boron depletion and systemic nutrient redistribution as key physiological features associated with embryo abortion, accompanied by reduced carbon-related quality traits in aborted fruits.

### 3.3 Transcriptomic profiling reveals broad metabolic and signaling reprogramming and repression of chloroplast-associated programs

RNA-seq generated high-quality datasets across biological replicates. After filtering, 45.16 Gb of clean bases were retained, with clean read ratios ranging from 94.00% to 96.13% and Q30 values from 91.35% to 92.09%. Clean reads mapped to the longan reference genome with overall alignment rates of 85.48–90.98%, uniquely mapped reads of 78.66–83.83%, and multi-mapped reads of 6.82–7.24%, indicating good reference compatibility and low ambiguity in read assignment (**Supplementary Table S2**).

**Figure 3:**
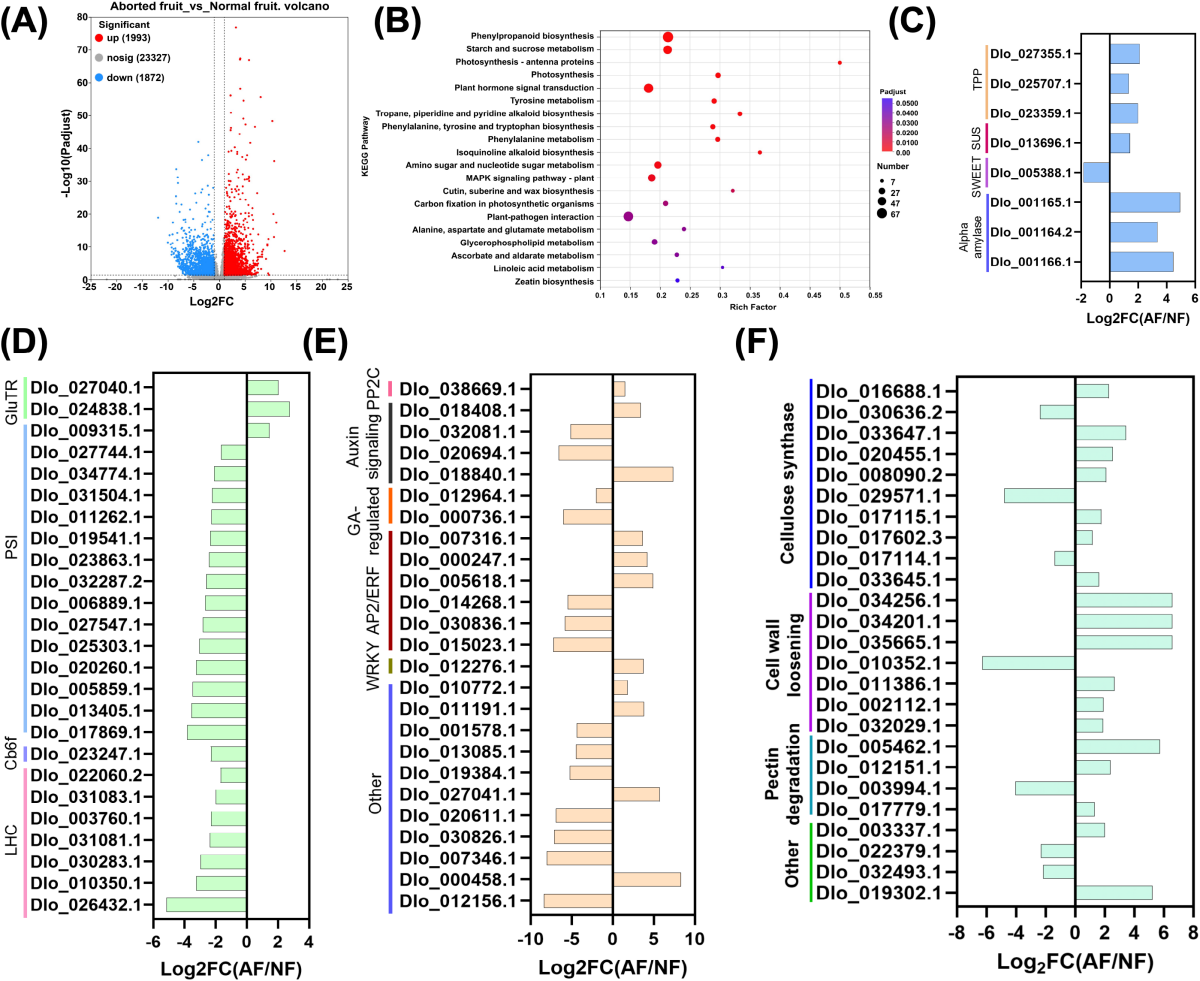
Transcriptomic differences between aborted-fruit embryos and normal-fruit embryos and expression changes in representative pathways/genes. **(A)** Volcano plot of differentially expressed genes (DEGs) identified by DESeq2 for AF vs NF embryos. Red dots indicate upregulated genes in AF, blue dots indicate downregulated genes in AF, and gray dots indicate non-significant genes. Numbers of genes in each class are shown (Up: 1,993; Down: 1,872; Not significant: 23,327); **(B)** KEGG enrichment bubble plot of DEGs. The x-axis shows the rich factor, bubble size represents the number of enriched genes, and bubble color indicates adjusted significance (Padj/FDR); **(C)** Expression changes of representative genes involved in carbohydrate supply, including starch mobilization, sucrose cleavage (e.g., sucrose synthase), trehalose/T6P pathway enzymes, and sugar transport, presented as Log2FC (AF/NF); **(D)** Expression changes of chloroplast/photosynthesis-related modules, including tetrapyrrole/chlorophyll biosynthesis, photosystem components (PSI/PSII subunits), cytochrome b6f complex, light-harvesting complex proteins (LHC; chlorophyll a/b-binding proteins), carotenoid/xanthophyll cycle genes, and Calvin/PPP link enzymes, shown as Log2FC (AF/NF); **(E)** Expression changes of genes associated with hormone signaling and regulatory factors, including auxin transport/response (e.g., auxin efflux carriers and auxin-induced proteins), ABA signaling components, GA-responsive regulators, and transcription factors involved in stress/hormone crosstalk (e.g., WRKY and AP2/ERF), shown as Log2FC (AF/NF); **(F)** Expression changes of cell wall–associated genes relevant to cell wall integrity/remodeling, including cellulose biosynthesis (CESA/cellulose synthase), cell-wall loosening (expansin), pectin modification/degradation (pectate lyase and related enzymes), and wall structural/signaling glycoproteins, shown as Log2FC (AF/NF). For panels **(C–F)**, genes were selected from significantly changed candidates within the indicated functional modules; positive Log2FC values indicate higher expression in AF, whereas negative values indicate higher expression in NF.

Reference-guided assembly identified 31,138 expressed genes (27,192 annotated and 3,946 newly predicted) and 52,175 transcripts. Differential expression analysis detected 3,865 DEGs between AF and NF embryos, including 1,993 upregulated and 1,872 downregulated genes in AF, while 23,327 expressed genes did not meet significance thresholds (**Figure 3A**). The volcano distribution showed extensive transcriptional divergence, with both strong induction and repression signals in AF embryos.

KEGG enrichment of DEGs revealed coordinated reprogramming spanning metabolism, signaling, and structural development (**Figure 3B**). Enriched pathways included phenylpropanoid biosynthesis and starch and sucrose metabolism, together with plant hormone signal transduction and MAPK signaling. Additional enriched terms captured amino sugar and nucleotide sugar metabolism, carbon metabolism, cutin/suberin/wax biosynthesis, plant–pathogen interaction, and several specialized metabolic routes. Notably, photosynthesis-related categories (photosynthesis; photosynthesis–antenna proteins; carbon fixation in photosynthetic organisms) also appeared among enriched pathways, largely driven by widespread downregulation of chloroplast-associated genes.

Candidate-level profiles clarified the directionality of key modules (**Figures 3C–F**). For carbohydrate supply and signaling (**Figure 3C**), aborted embryos showed strong induction of starch mobilization genes, including α-amylases (Dlo_001165.1, Dlo_001166.1, Dlo_001164.2), and trehalose/T6P-related components (Dlo_027355.1, Dlo_023359.1, Dlo_025707.1), together with increased sucrose cleavage capacity (sucrose synthase Dlo_013696.1). In contrast, a SWEET sugar transporter (Dlo_005388.1) was reduced, indicating enhanced internal mobilization but altered interfacial sugar transport. Chloroplast-associated programs were predominantly repressed (**Figure 3D**). Light-harvesting chlorophyll a/b-binding proteins were strongly downregulated (e.g., Dlo_026432.1, Dlo_030283.1, Dlo_010350.1, Dlo_031081.1), accompanied by broad suppression of photosystem components (PSI: Dlo_017869.1, Dlo_013405.1, Dlo_020260.1; PSII: Dlo_005859.1, Dlo_027547.1, Dlo_032287.2), cytochrome b6/f-related factors (Dlo_023247.1), and carotenoid-related genes (Dlo_006717.1). Only a few pigment/tetrapyrrole-linked genes showed induction (e.g., Dlo_024838.1, Dlo_027040.1), but the dominant trend across chloroplast modules was coordinated repression. Hormone and MAPK regulatory genes displayed extensive rewiring (**Figure 3E**). Auxin transport components, particularly auxin efflux carriers (Dlo_020694.1, Dlo_032081.1), were markedly reduced, whereas stress-associated regulatory nodes showed activation, including induction of WRKY (Dlo_012276.1) and changes in AP2/ERF and ABA-related PP2C modules (e.g., Dlo_005618.1, Dlo_015023.1, dlo_038669.1). GA-related regulators were generally reduced (e.g., Dlo_000736.1, Dlo_012964.1), consistent with growth restraint during abortion. Cell wall–associated genes showed a pronounced imbalance between remodeling and synthesis (**Figure 3F**). Pectin degradation enzymes were induced (Dlo_005462.1, Dlo_012151.1), while one pectate lyase was repressed (Dlo_003994.1). Expansins displayed strong bidirectionality, with several members induced (Dlo_034256.1, Dlo_034201.1, Dlo_035665.1, Dlo_011386.1) but another sharply repressed (Dlo_010352.1), indicating divergent wall-loosening programs. Cellulose synthase genes were split, with multiple members induced (Dlo_033647.1, Dlo_020455.1, Dlo_016688.1) but one key cellulose synthase strongly downregulated (Dlo_029571.1), supporting a dysregulated cell wall program associated with embryo abortion.

Together, these transcriptomic results demonstrate that AF embryos undergo broad metabolic and signaling reprogramming characterized by enhanced carbohydrate mobilization and stress-regulatory responses, coupled with repression of chloroplast-associated programs and imbalanced cell wall remodeling. qRT-PCR validation of mechanism-anchored candidates further supported the reliability of the RNA-seq quantification (Section 3.6).

### 3.4 Proteomic profiling reveals extensive protein down-accumulation and functional remodeling in aborted embryos

**Figure 4:**
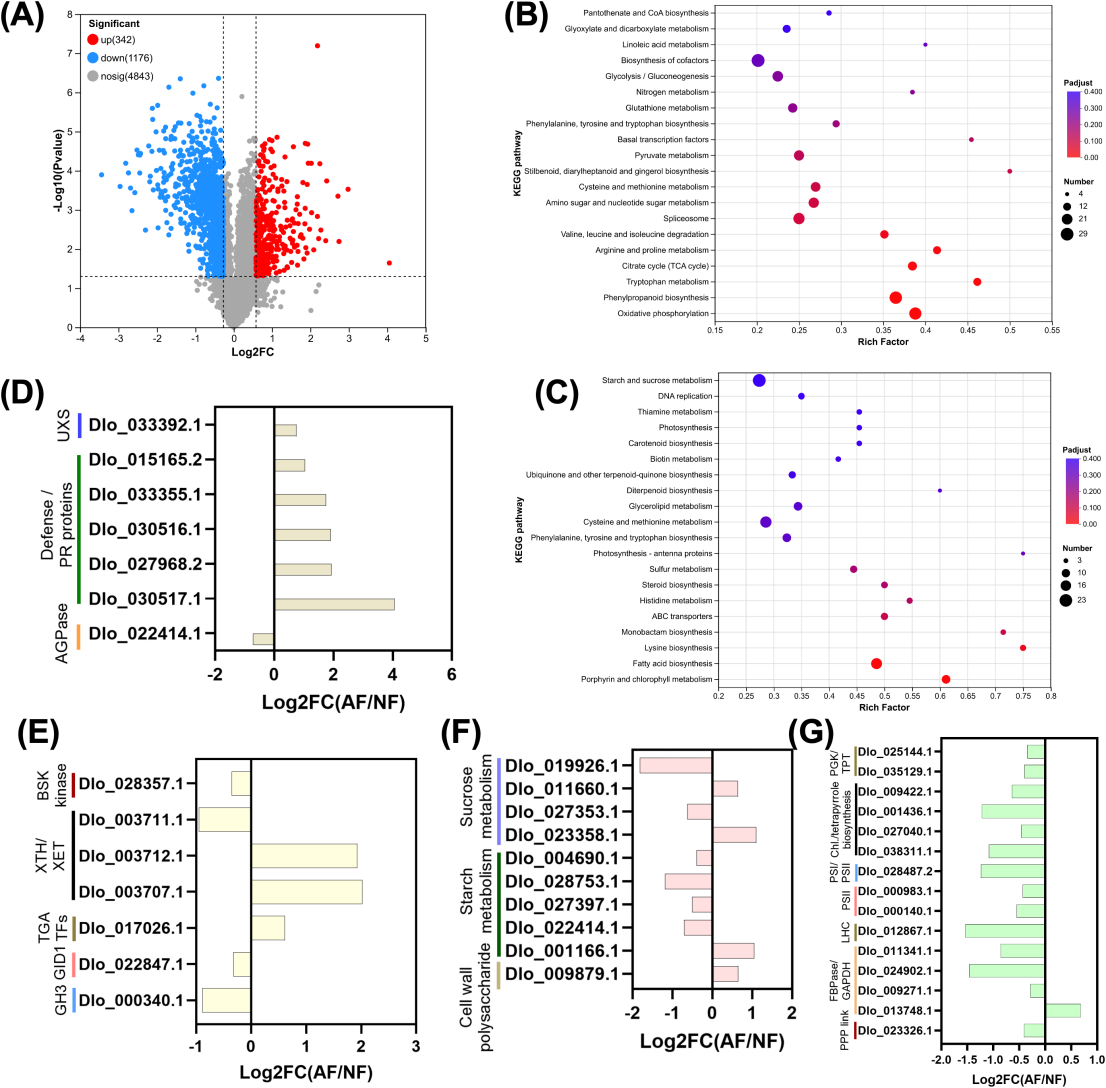
Transcriptomic divergence between aborted fruit and normal fruit embryos and pathway-level changes associated with embryo abortion. **(A)** Volcano plot of differentially expressed genes (DEGs) for AF vs NF, showing upregulated genes in AF (red), downregulated genes in AF (blue), and non-significant genes (gray); the numbers of upregulated (1,943), downregulated (1,872), and non-significant genes (23,327) are indicated in the legend. **(B, C)** KEGG enrichment bubble plots for DEGs upregulated in AF **(B)** and downregulated in AF **(C)**; the x-axis denotes the rich factor, bubble size represents the number of genes mapped to each pathway, and bubble color indicates adjusted significance (Padj). **(D)** Expression changes of representative DEGs related to defense/cell-wall precursor metabolism (e.g., chitinases/endochitinases, UDP-xylose synthesis) and starch precursor synthesis (AGPase), shown as Log2FC (AF/NF). **(E)** Expression changes of representative hormone-associated regulators and signaling components (e.g., GH3, GID1b, BSK1, TGA, and XTH family members), shown as Log2FC (AF/NF). **(F)** Expression changes of carbohydrate metabolism and cell-wall polysaccharide turnover genes, including sucrose metabolism (invertases, sucrose synthase), starch metabolism (amylase/starch synthases/branching enzyme, AGPase), and cell-wall glucan remodeling (endo-β-1,4-glucanase), shown as Log2FC (AF/NF). **(G)** Expression changes of chloroplast/photosynthesis-related modules, including Calvin/PPP-linked carbon metabolism (e.g., transketolase), tetrapyrrole/chlorophyll biosynthesis (e.g., GluTR, ALAD, porphobilinogen deaminase, Mg-porphyrin cyclase), photosystem/OEC components, and light-harvesting chlorophyll a/b-binding proteins (LHC), shown as Log2FC (AF/NF). Positive values indicate higher expression in AF, whereas negative values indicate higher expression in NF.

TMT-based quantitative proteomics detected widespread protein-level differences between NF and AF embryos. In total, 1,518 differentially accumulated proteins (DAPs) were identified, of which 342 increased and 1,176 decreased in AF relative to NF (P < 0.05; criteria as defined in Methods), indicating a strong global tendency toward reduced protein abundance in aborted embryos (**Figure 4A**). The magnitude distribution of fold changes further showed that down-accumulated proteins dominated the proteomic response, consistent with a broad suppression of biosynthetic capacity during embryo abortion.

KEGG enrichment of increased DAPs highlighted pathways associated with energy production and stress-adaptive metabolism (**Figure 4B**), including oxidative phosphorylation, citrate cycle (TCA cycle), amino acid metabolism and degradation (e.g., valine/leucine/isoleucine degradation; arginine and proline metabolism; tryptophan metabolism), phenylpropanoid biosynthesis, glycolysis/gluconeogenesis, glutathione metabolism, and related cofactor and nitrogen metabolism pathways. By contrast, KEGG enrichment of decreased DAPs was dominated by anabolic and plastid-associated processes (**Figure 4C**), including porphyrin and chlorophyll metabolism, fatty acid biosynthesis, photosynthesis and photosynthesis–antenna proteins, carotenoid biosynthesis, terpenoid-quinone pathways (ubiquinone and related), and additional biosynthetic and transport modules such as ABC transporters.

Candidate-level protein profiles further clarified the directionality of key functional modules (**Figures 4D–G**). In cell wall precursor and defense-related proteins (**Figure 4D**), a UDP-glucuronic acid decarboxylase/UXS (Dlo_033392.1) increased in AF, together with multiple chitinases and endochitinases representing defense/PR proteins (Dlo_015165.2, Dlo_033355.1, Dlo_030516.1, Dlo_027968.2, Dlo_030517.1), whereas a key starch biosynthesis enzyme AGPase large subunit (Dlo_022414.1) decreased. In hormone-linked proteins (**Figure 4E**), the brassinosteroid-associated kinase BSK1-like protein (Dlo_028357.1) decreased, xyloglucan endotransglucosylase/hydrolases displayed mixed directionality (Dlo_003707.1 and Dlo_003712.1 increased, Dlo_003711.1 decreased), a TGA transcription factor (Dlo_017026.1) increased, and two canonical hormone signaling components were reduced, including the GA receptor GID1b (Dlo_022847.1) and the auxin-conjugating enzyme GH3 (Dlo_000340.1).

Proteins involved in carbohydrate metabolism showed a pattern consistent with enhanced mobilization but weakened storage biosynthesis (**Figure 4F**). Sucrose metabolism proteins were bidirectional, with a sucrose synthase (Dlo_019926.1) decreased, while invertases included both increases (cell wall-bound invertase Dlo_011660.1 and soluble acid invertase 2 Dlo_023358.1) and a decrease (acid invertase Dlo_027353.1). Starch metabolism proteins were dominated by reductions in biosynthetic enzymes, including starch synthases (Dlo_027397.1, Dlo_004690.1) and a branching enzyme (Dlo_028753.1), while a starch-degrading α-amylase (Dlo_001166.1) increased. A cell wall polysaccharide-modifying enzyme, endo-(1,4)-β-D-glucanase (Dlo_009879.1), also increased.

Chloroplast-associated programs were broadly suppressed at the protein level (**Figure 4G**). Light-harvesting and photosystem-related proteins were reduced, including a chlorophyll a/b-binding protein (Dlo_012867.1), a photosystem reaction center IV family protein (Dlo_028487.2), and oxygen-evolving enhancer proteins (Dlo_000983.1, Dlo_000140.1). Tetrapyrrole and chlorophyll biosynthesis enzymes were also decreased, including glutamyl-tRNA reductase (Dlo_027040.1), δ-aminolevulinic acid dehydratase (Dlo_038311.1), porphobilinogen deaminase (Dlo_009422.1), and magnesium-protoporphyrin IX monomethyl ester cyclase (Dlo_001436.1). Calvin/PPP-associated enzymes were predominantly reduced, including chloroplastic phosphoglycerate kinase (Dlo_035129.1), triosephosphate isomerase (Dlo_025144.1), chloroplastic FBPase (Dlo_024902.1), chloroplastic GAPDH (Dlo_011341.1), and transketolase (Dlo_023326.1), whereas a cytosolic GAPDH (Dlo_013748.1) increased.

Together, **Figure 4** shows that AF embryos exhibit a proteomic landscape characterized by dominant down-accumulation of proteins and coordinated functional remodeling. The increased DAPs are enriched for energy and stress-adaptive metabolism, while decreased DAPs concentrate in chloroplast-associated pathways, lipid and pigment biosynthesis, and other anabolic functions, consistent with a shift away from growth-supporting programs during embryo abortion.

### 3.5 Integrated transcriptome–proteome association analysis reveals strong concordance and highlights key embryo-abortion–related pathways

**Figure 5:**
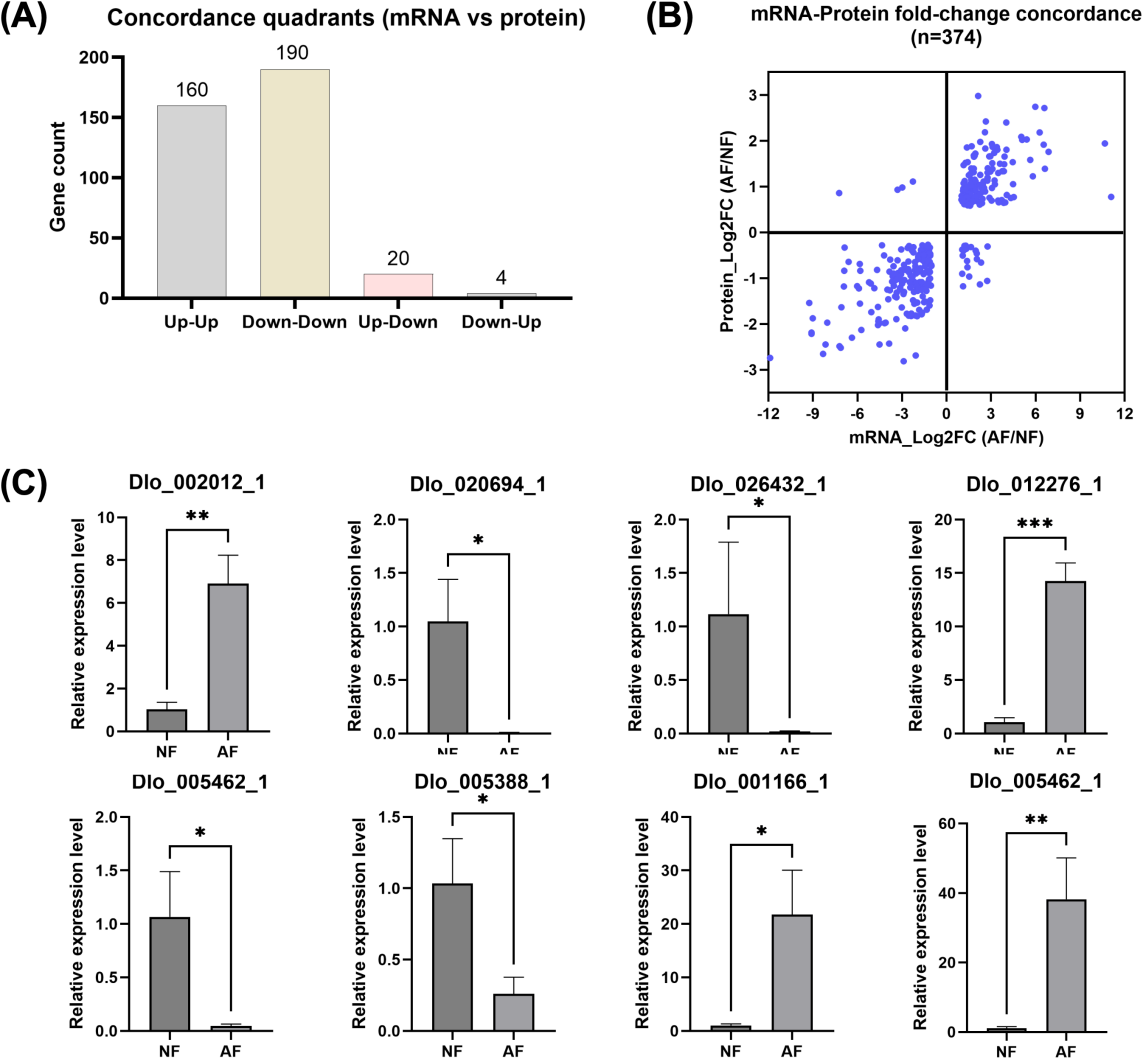
Integration of transcriptomic and proteomic fold changes and qRT–PCR validation of representative candidate genes associated with embryo abortion in longan. **(A)** Distribution of shared genes (n = 374) across concordance quadrants between mRNA and protein regulation; **(B)** Scatter plot showing mRNA–protein fold-change concordance for the shared gene set. Horizontal and vertical lines indicate Log2FC = 0, separating the four regulation quadrants; **(C)** qRT–PCR validation of representative candidates across mechanistic modules, comparing embryos from normal fruit (NF) and aborted fruit (AF). Transcript levels were normalized to ACTIN and calculated using the 2^-ΔΔCt^ method; bars indicate mean ± SD (biological replicates), and significance is denoted as *P < 0.05, **P < 0.01, and ***P < 0.001.

To evaluate the consistency between transcriptional and translational regulation during embryo abortion, we integrated DEGs and DAPs using shared gene identifiers. A total of 374 overlapping genes/proteins were identified across the transcriptome and proteome datasets (**Supplementary Table S3**). Overall, mRNA and protein fold changes were highly consistent, showing a strong positive correlation between Log2FC(mRNA) and Log2FC(protein) (Pearson r = 0.818; Spearman ρ = 0.818). Quadrant-based concordance analysis further indicated that 350/374 (93.6%) of the overlapping features displayed concordant directional changes, including 160 Up–Up and 190 Down–Down genes/proteins. Only 24/374 (6.4%) features were discordant, comprising 20 Up–Down and 4 Down–Up cases (**Figures 5A, B**; **Supplementary Table S4**).

Pathway-level integration based on the 374 overlapping features (**Supplementary Table S5**) identified several modules that were consistently altered at both mRNA and protein levels. Phenylpropanoid biosynthesis was the most represented pathway among the overlap set (17 genes, all concordant; predominantly Up–Up), indicating coordinated activation of secondary metabolism at both layers. Amino sugar and nucleotide sugar metabolism was also prominent (12 genes, all concordant; mostly Up–Up), consistent with remodeling of sugar precursors linked to cell-wall–related processes. Starch and sucrose metabolism contained 10 overlap genes with complete directional concordance, but the module showed a mixed internal structure, with induction of mobilization/unloading enzymes such as α-amylase (Dlo_001166.1) and invertase-like components alongside coordinated reductions in several biosynthesis/traNFer steps, yielding an overall negative median shift for this pathway at both mRNA and protein levels.

Developmental signaling modules were also represented within the overlap set, including plant hormone signal transduction (5 genes, all concordant) and MAPK signaling pathway–plant (5 genes, all concordant). These pathways included stress- and signaling-associated nodes such as an XTH-like glycoside hydrolase (e.g., Dlo_003707.1/Dlo_003712.1) and ROS-related components in the MAPK module (e.g., RBOH/cytochrome b245 heavy chain genes Dlo_006450.1 and Dlo_002012.1), supporting extensive regulatory rewiring during abortion.

In contrast, chloroplast-associated programs within the overlap set were strongly biased toward repression. Photosynthesis (2 genes) and photosynthesis–antenna proteins (2 genes) were uniformly Down–Down, while carbon fixation in photosynthetic organisms (6 genes) was dominated by Down–Down changes with a small compensatory subset, resulting in an overall negative shift at both mRNA and protein levels. Porphyrin and chlorophyll metabolism (3 genes) also trended downward (mostly Down–Down), consistent with repression of pigment/tetrapyrrole-associated processes. Finally, redox/energy-related modules within the overlap set showed coherent suppression, including glutathione metabolism (6 genes, all concordant and Down–Down) and glycolysis/gluconeogenesis (7 genes, all concordant with a negative median shift), supporting altered redox buffering and central energy metabolism in aborted embryos.

### 3.6 qRT-PCR validation of embryo-abortion–related candidate genes

To validate the RNA-seq quantification and support candidate prioritization, eight embryo-abortion–related genes were selected to represent the five mechanistic axes identified by integrative multi-omics analysis: B/cell wall integrity (Dlo_005462.1, pectate lyase; Dlo_029571.1, cellulose synthase), carbon supply (Dlo_001166.1, α-amylase; Dlo_005388.1, SWEET transporter), hormone and MAPK regulation (Dlo_020694.1, auxin efflux carrier; Dlo_012276.1, WRKY transcription confirmed by RNA-seq), chloroplast program (Dlo_026432.1, chlorophyll a/b-binding protein), and redox/ROS signaling (Dlo_002012.1, respiratory burst oxidase homolog, RBOH) (**Supplementary Table S1**).

qRT-PCR assays using ACTIN as the reference gene supported RNA-seq trends for the selected candidates, showing consistent directional changes between AF and NF embryos across the five axes (**Figure 5C**; **Supplementary Table S1**). Candidates representing carbon mobilization and stress signaling, such as α-amylase, WRKY and RBOH, showed higher transcript levels in AF embryos, whereas candidates related to sugar transport/sink establishment (SWEET), auxin transport (auxin efflux carrier), cellulose synthesis (CESA) and chloroplast-associated programs (chlorophyll a/b-binding protein Dlo_026432.1) showed reduced transcript levels in AF embryos.

## 4 Discussion

This study integrates embryo-focused physiology with transcriptomics and quantitative proteomics to establish a coherent mechanistic framework for longan embryo abortion. Across datasets, five axes repeatedly provided the best explanatory power. These axes included embryonic boron deficiency with disrupted cell wall integrity, perturbed carbon supply with carbohydrate reprogramming, hormone and MAPK regulatory rewiring, repression of chloroplast-associated programs including photosystems and pigment metabolism, and redox and energy imbalance. The transcriptome–proteome association for shared genes was strongly positive, indicating that transcriptional regulation is a major driver at the critical abortion stage, while a smaller discordant subset likely reflects post-transcriptional buffering and protein stability control.

### 4.1 Boron deficiency and nutrient misallocation form an early physiological basis for embryo abortion

Boron can directly translate micronutrient status into embryo viability because its canonical biochemical function is the formation of borate diester cross-links that dimerize rhamnogalacturonan-II (RG-II) domains and stabilize primary cell wall architecture and cell–cell cohesion (Kobayashi et al., 1996; O'Neill et al., 2004; Funakawa and Miwa, 2015; Begum and Fry, 2023; Hays et al., 2025). In our dataset, the embryo displayed a distinctive boron depletion signature relative to maternal tissues, consistent with allocation failure rather than uniform deficiency. The opposite trends of boron (increased in leaves/pericarps but decreased in embryos) are consistent with tissue-specific partitioning and transport constraints. Boron is predominantly delivered via the xylem and often shows limited phloem mobility; therefore, transpiring maternal tissues (e.g., leaves) and maternal fruit tissues can accumulate boron even when boron delivery to the low-transpiring filial embryo is restricted (Miwa and Fujiwara, 2010). In addition, impaired maternal–filial transfer across the seed interfaces and reduced embryo sink strength during the abortion window could further limit boron import into embryos, yielding an apparent “maternal sufficiency–filial depletion” pattern. Accordingly, we interpret embryonic boron depletion as evidence of allocation/transfer limitation at the maternal–filial interface rather than a uniform whole-plant boron deficiency. This pattern is biologically plausible because boron homeostasis depends on coordinated uptake and long-distance transport mediated by NIP aquaporin channels and BOR exporters, and disruption of these transport modules can cause severe reproductive defects under low boron conditions (Takano et al., 2006, Takano et al., 2010; Takada et al., 2014; Zhou et al., 2025). Within the embryo, coupled transcriptome and proteome evidence pointed to extensive remodeling of cell wall-related processes, including pectin modification or degradation and cellulose or hemicellulose remodeling. A notable feature was the coexistence of strongly induced wall-loosening or wall-degradation factors such as pectate lyases and expansins with suppressed members of wall synthesis machinery, including selected cellulose synthase components. This pattern is consistent with an unbalanced, stress-driven wall remodeling state rather than coordinated wall construction. A parsimonious interpretation is a self-reinforcing failure mode in which boron shortage weakens RG-II cross-linking and pectin cohesion, triggers compensatory remodeling programs, and then proceeds in a disordered manner that further compromises tissue integrity and blocks organized embryo growth.

Boron deficiency can also reshape downstream pathways by altering the physical and signaling properties of the wall–plasma membrane continuum. Cell wall integrity signaling interfaces with Ca^2+^-dependent pathways, MAPK cascades, and hormone networks, enabling wall defects to rapidly reprogram development (Verma et al., 2021; Foyer et al., 2022). This supports a model in which boron-linked wall instability provides an upstream trigger capable of propagating across the other axes identified here. Future work should directly test whether boron supplementation can partially restore embryo development and reduce abortion incidence, thereby validating causality.

### 4.2 Carbon supply disturbance triggers embryonic catabolic reprogramming and impaired sink establishment

A central feature of embryo abortion across species is inadequate carbon delivery to filial tissues during rapid early growth. This is often expressed as reduced sink strength and diversion of carbon toward stress and defense programs (Saini et al., 2025). In developing seeds, sucrose traNFer from maternal tissues to the embryo requires coordinated apoplastic unloading and membrane transport, mediated by SWEET transporters and related importer systems. Downstream sucrose cleavage and metabolite partitioning then enable embryo expansion and storage synthesis (Chen et al., 2015; Ji et al., 2022; Pegler et al., 2023). In our study, starch and sucrose metabolism was extensively altered at both mRNA and protein levels. The directionality of these changes suggests that aborted embryos do not simply receive more sugar. Instead, the profile is consistent with carbon stress and metabolic compensation. Starch-mobilizing enzymes such as alpha-amylases and sucrose-cleavage enzymes such as sucrose synthase tended to increase, whereas at least one SWEET transporter showed suppression. This combination supports a mobilization without effective delivery scenario in which embryos activate internal catabolic programs to sustain respiration and survival, but interfacial sugar flux into the embryo remains limiting.

The trehalose and T6P module reinforces this interpretation. Changes in trehalose-phosphate pathway enzymes are widely recognized as sensitive indicators of carbon status signaling and growth restraint. These nodes frequently act through SnRK1-centered networks to prioritize survival over biomass accumulation under stress. The reprogramming observed for trehalose-related components aligns with a carbon-starvation adaptive state rather than a growth-permissive sink state. Together, these carbohydrate signatures provide a mechanistic explanation for the phenotypic endpoint. Insufficient assimilate availability prevents coordinated cell division, wall synthesis, and reserve deposition and culminates in embryo growth arrest.

### 4.3 Hormone and MAPK rewiring integrates stress and development

Embryogenesis depends on tightly regulated hormone gradients and signal integration. Auxin transport and response are particularly critical for embryo patterning and organogenesis, and disruption of auxin efflux carriers or auxin-responsive modules commonly produces severe embryo defects (Verma et al., 2021). Our multi-omics results revealed pronounced shifts in hormone signaling components, including downregulation of auxin transport and response factors and concomitant changes in other hormone-associated regulators such as PP2C-related ABA signaling components and GA-linked regulators. This profile is consistent with growth restraint in aborted embryos. Auxin-related patterning and growth signals are diminished, while ABA-associated stress programs rise and GA-associated growth programs are curtailed, together favoring developmental arrest.

MAPK signaling and stress-responsive transcription factors provide a mechanistic coupling between upstream wall or nutrient perturbation and downstream gene reprogramming. WRKY induction, frequently downstream of MAPK and ROS signals, was prominent in aborted embryos and is consistent with activation of defense-like programs and degeneration trajectories. Evidence from related fruit systems supports a causal role of ROS in seed abortion. In litchi, DNA methylation-mediated ROS production has been implicated as a contributor to seed abortion (Xie et al., 2024). In our dataset, the presence of ROS-related modules, including RBOH-associated signals at the protein level, supports a model in which oxidative signaling acts both as a stress amplifier and as a regulatory switch that reshapes hormone outputs and reinforces growth arrest.

### 4.4 Repression of chloroplast-associated programs reflects impaired plastid function

A highly consistent signature in aborted embryos was repression of chloroplast/plastid-associated modules, including photosystem components, light-harvesting proteins, and tetrapyrrole/pigment biosynthesis enzymes. While embryos are not major photosynthetic organs, developing embryos contain differentiating plastids that serve as essential biosynthetic hubs for amino acids, fatty acids, isoprenoids, and hormone precursors. Therefore, we interpret these signals primarily as impaired plastid/chloroplast biogenesis and plastid-associated energy/redox metabolism rather than “leaf-like” photosynthesis per se (Chen et al., 2018; Li et al., 2021; Dupouy et al., 2022). Notably, although embryos were dissected under a stereomicroscope, minor maternal tissue carryover cannot be completely excluded in field-derived samples; thus, we interpret the chloroplast-related repression conservatively.

This interpretation is strengthened by coupled repression of pigment and tetrapyrrole synthesis factors and photosystem assembly or maintenance components, which together indicate compromised plastid development and thylakoid-associated processes. Such dysfunction would reduce the embryo’s ability to sustain anabolic metabolism and redox buffering during a stage that normally requires rapid biosynthesis. Plastid redox signaling is also tightly coupled to nuclear gene expression, which provides a route by which plastid impairment can drive broad transcriptional remodeling overlapping with stress and hormone pathways (Geigenberger, 2014; Foyer et al., 2022). In the working model, chloroplast program repression functions as both a consequence of upstream stress and a contributor to the irreversible progression toward abortion by limiting biosynthetic capacity and redox homeostasis.

### 4.5 Redox and energy imbalance reinforce embryo degeneration and abortion

Embryo abortion often culminates in a shift from anabolic growth to catabolic maintenance, accompanied by oxidative stress and altered energy metabolism. In our integrated data, multiple redox and energy indicators were consistent with this shift. Oxidative phosphorylation and central carbon metabolism modules were remodeled, and antioxidant and redox-related functions were implicated within broader reprogramming. These changes are consistent with a state in which embryos attempt to maintain ATP and redox homeostasis under constrained carbon supply and compromised plastid function but ultimately fail to preserve developmental progression. Identification of ROS-linked nodes and redox-related proteins provides plausible mechanistic links to downstream cellular outcomes such as membrane damage, loss of organelle integrity, and cell death-like processes, aligning with the observed embryo degeneration phenotype (Geigenberger, 2014; Foyer et al., 2022; Xie et al., 2024).

**Figure 6:**
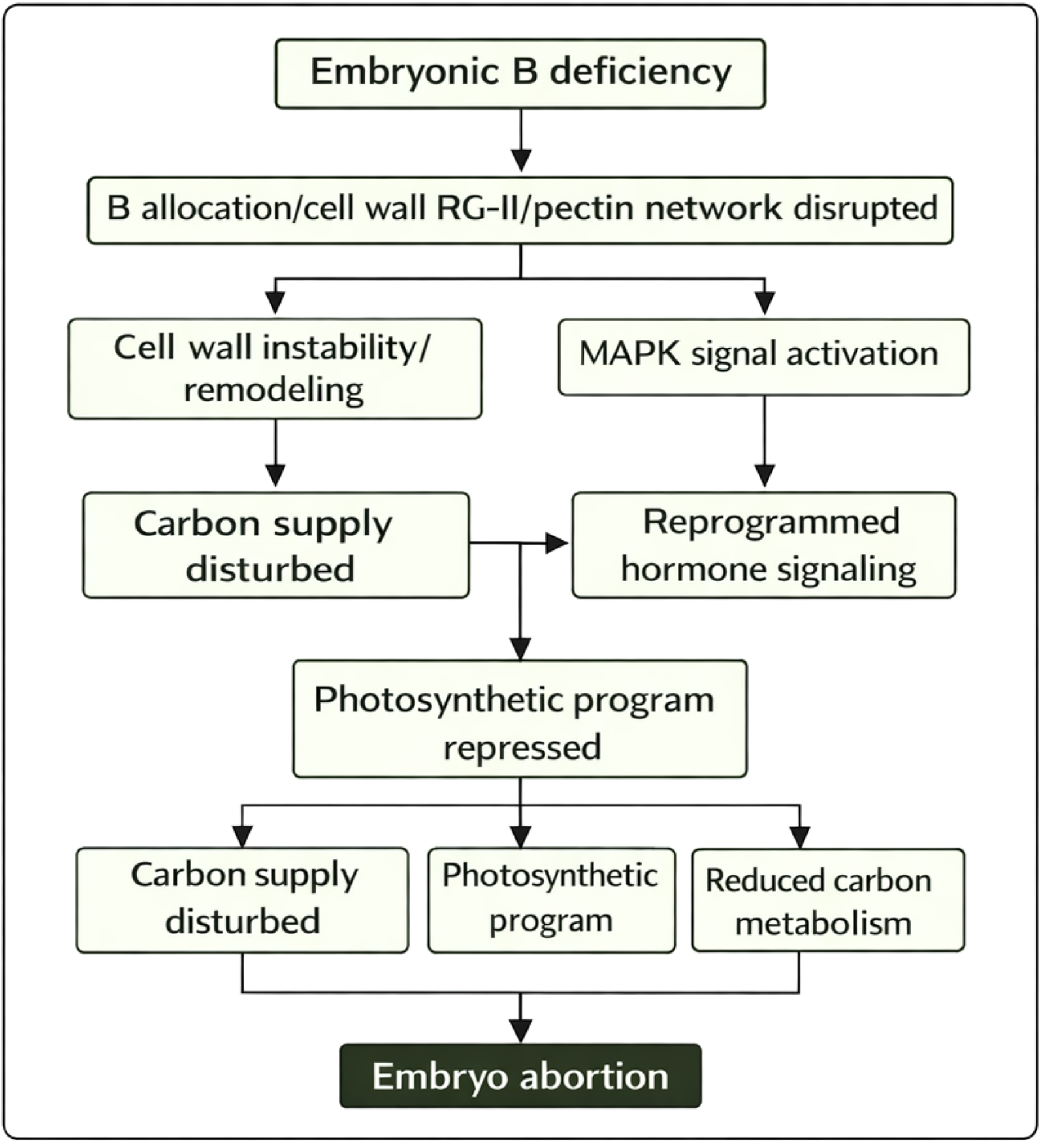
Proposed model of longan embryo abortion.

The results support a working model in which embryo-associated B depletion and altered allocation are associated with weakened RG-II/pectin-mediated wall cohesion and stress-driven wall remodeling (**Figure 6**), which may destabilize the maternal–embryo interface and reduce effective sink establishment. The embryo exhibits carbohydrate mobilization and carbon-status signaling yet fails to sustain growth. In parallel, hormone and MAPK pathways are rewired, with ROS signaling acting as an amplifier and integrator of stress, and plastid-associated programs are repressed, further constraining biosynthetic and redox capacity. Because our measurements capture a single critical window, we cannot fully resolve whether B depletion precedes abortion or is a consequence of early degeneration; longitudinal sampling and targeted B manipulation will be required to establish temporal causality.

## 5 Conclusion

By integrating stage-resolved phenotyping and nutrient profiling with embryo-focused transcriptomics and quantitative proteomics, this study delineates a coherent multi-layer mechanism underlying embryo abortion in longan. Aborted embryos exhibited a distinctive nutrient-partitioning phenotype, most notably severe embryonic boron depletion, together with compromised carbon status reflected by reduced sugar-associated traits. Multi-omics profiling identified a coordinated molecular program characterized by extensive cell wall remodeling linked to boron/pectin (RG-II) integrity, disrupted carbohydrate allocation with compensatory starch and sucrose mobilization, broad hormone–MAPK/WRKY reprogramming consistent with stress-driven developmental restraint, and pronounced repression of chloroplast-associated programs including photosynthetic light reactions and pigment/tetrapyrrole metabolism, accompanied by redox and energy imbalance. Cross-omics integration further showed high concordance between transcript and protein fold changes, indicating that transcriptional reprogramming is largely transmitted to the protein level during the critical abortion stage. Together, these physiological and molecular disruptions converge on embryo growth arrest and subsequent degeneration. The candidate genes and pathway modules prioritized here provide practical markers and mechanistic targets for diagnosing abortion risk and for developing nutritional and genetic strategies to improve seed development and fruit set in longan.

## Data Availability

The datasets presented in this study can be found in online repositories. The transcriptome data have been deposited in the NCBI SRA under BioProject PRJNA1419010 (SRA: PRJNA1419010) and the proteomics data have been deposited in the iProX/ProteomeXchange repository with accession IPX0015639000.

## Supplementary material

**Supplementary Figure S1** Soluble sugar and vitamin C contents in normal fruits and aborted fruits. **(A)** Soluble sugar content (mg g^-^¹ fresh weight, FW). **(B)** Vitamin C content (μg g^-^¹ FW). Bars represent mean ± SD (n = 3 biological replicates). Different lowercase letters above bars indicate significant differences between NF and AF (P < 0.05; independent-samples t-test).
