# Supplementary figures and images for: Integrated physiological, multi-omics analyses reveal key factors underlying seed abortion in Dimocarpus longan
Source: Front Plant Sci. 2026 Mar 2;17:1778131. doi: 10.3389/fpls.2026.1778131 (PMC12990130; doi:10.3389/fpls.2026.1778131)

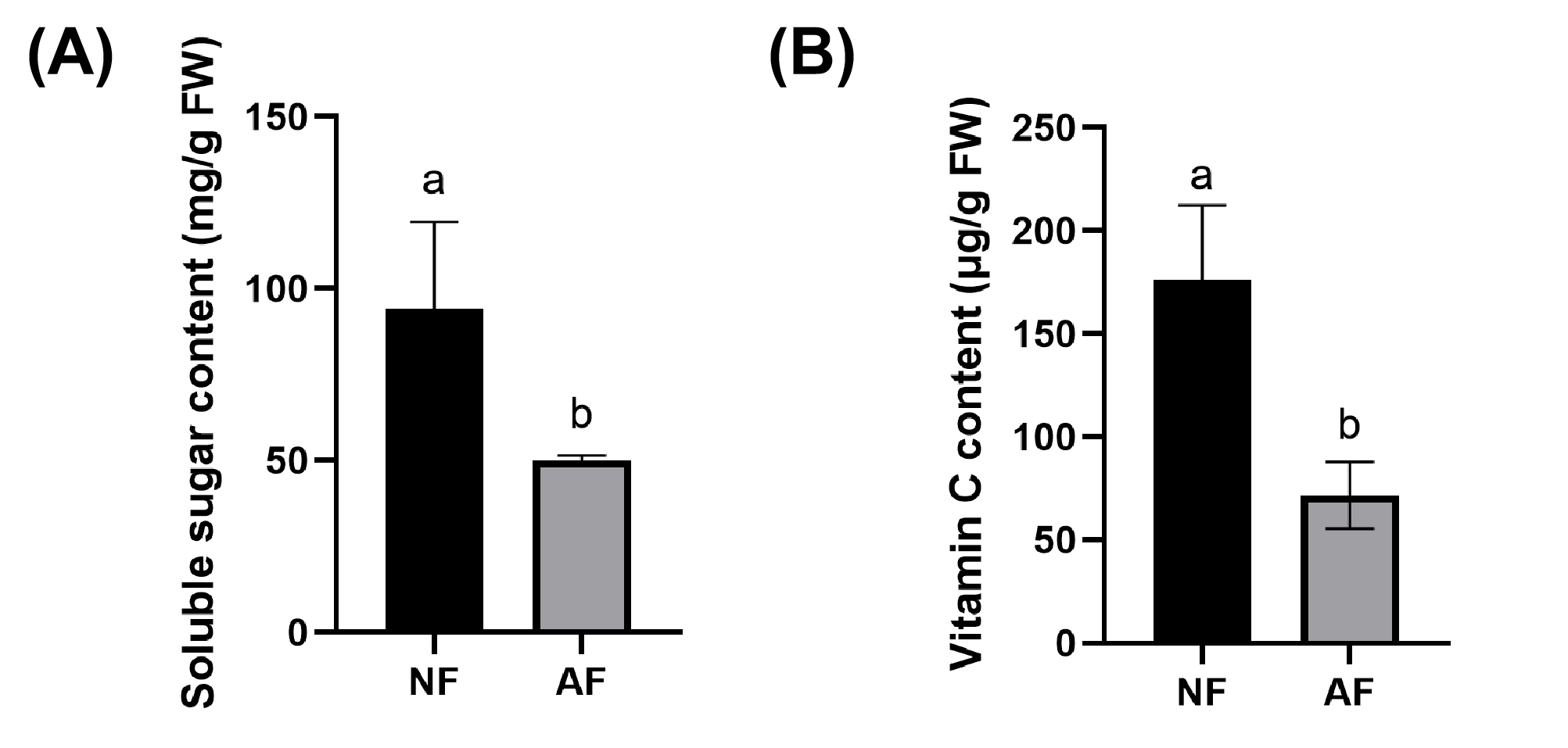

Supplement: Supplementary file 1 — Supplementary Figure S1 Soluble sugar and vitamin C contents in normal fruits and aborted fruits. (A) Soluble sugar content (mg g-¹ fresh weight, FW). (B) Vitamin C content (μg g-¹ FW). Bars represent mean ± SD (n = 3 biological replicates). Different lowercase letters above bars indicate significant differences between NF and AF (P < 0.05; independent-samples t-test). [file Image1.tif]
